# Influence of Solvent Evaporation Technique Parameters on Diameter of Submicron Lamivudine-Poly-ε-Caprolactone Conjugate Particles

**DOI:** 10.3390/nano9091240

**Published:** 2019-08-31

**Authors:** Tomasz Urbaniak, Witold Musiał

**Affiliations:** Department of Physical Chemistry and Biophysics, Pharmaceutical Faculty, Wroclaw Medical University, Borowska 211, 50-556 Wroclaw, Poland

**Keywords:** drug delivery, biodegradable polymers, drug-polymer conjugates, submicron particle preparation, solvent evaporation technique

## Abstract

The size of active pharmaceutical ingredient carrier is one of the key properties considered during design of submicron drug delivery systems. Particle diameter may determine drug biodistribution, cellular uptake, and elimination path. Solvent evaporation technique is a flexible method of particle preparation, in which various macromolecules and drugs may be employed. Parameters of emulsion obtained as first step of particle preparation are crucial in terms of particle size, drug loading, and morphology. The aim of the study was to investigate the influence of emulsion preparation parameters on diameter of resulting particles. Impact of surfactant type and concentration, homogenization time, homogenization rate, phase ratio, and conjugate concentration were evaluated. Model drug lamivudine was covalently bound to polymer and applied in solvent evaporation method in order to overcome issues related to drug loading and provide method-independent incorporation. Synthesized drug–polymer conjugate and obtained particles were evaluated via dynamic light scattering, chromatography, scanning electron microscopy, and spectroscopic methods. Covalent bonding between drug and polymeric chain was confirmed, estimated drug content per milligram of conjugate was 19 μg. Among employed colloid stabilizer, poly(vinyl alcohol) was proven to be most effective. Homogenization rate and surfactant concentration were identified as crucial parameters in terms of particle diameter control.

## 1. Introduction

### 1.1. Size-Dependent Carrier Internalization

Constantly discovered molecular and cellular targets pose new challenges in the field of pharmaceutical sciences focused on selective targeting strategies employing nano- and micro- particle systems. Cellular uptake, membrane crossing, and tissue penetration are all size-dependent phenomena. Therefore, particle diameter is one of the fundamental parameters considered during drug delivery system design [1]. The nature of interaction between particle surface and cellular membrane receptor is considered to be the main factor determining the internalization pathway. The number of engaged receptors, as well as the ligand-receptor affinity, are important factors influencing drug uptake. Undoubtedly, size of particle is the main parameter determining the surface area of particle-cell contact and the number of interacting receptors. Therefore, it significantly influences uptake mechanism and efficiency [2]. The following mechanisms are predominating ways of particle endocytosis with increasing particle diameter: caveolin-mediated (~50–80 nm), clathrin-mediated (~120 nm), macro-pinocytosis (~100–5 µm), and phagocytosis (~0.5–10 µm) [3,4]. Particle diameter, shape, surface physiochemical properties, protein corona composition, surface zeta potential and type of interacting cell determine efficiency of particles internalization by the cells [5]. Another important aspect of particle-based therapy influenced by size is the particle distribution in the body. In order to increase concentration of administered particles in tumors, enhanced permeability and retention effect can be exploited. Endothelial gap of blood vessels stimulated by tumors is larger, which promotes diffusion of macromolecular species [6]. Various size-selective barriers present in organs and between tissues and surrounding fluids are significantly influencing particle biodistribution [7]. Overwhelming percent of introduced particles is deposited in the spleen and liver. Introduction of smaller particles can increase their concentration in the brain due to the possibility of blood-brain barrier crossing, a significant part of injected micrometer sized particles tends to accumulate in lungs [8]. Particle shape is also factor which determines injected material accumulation in particular tissues, discoidal particles exhibited larger accumulation in most of organs with simultaneously reduced accumulation in liver in contrast to spheres. [9]. Spherical particles with diameters in range of tens of nanometers exhibit capability to accumulate in tumors, while slightly larger filamentous particles of same material aggregate in spleen [10].

### 1.2. Particles Uptake by Macrophages

In recent decades, a number of research groups have focused their attention on contribution of monocyte-derived immune cells to various diseases including obesity, fibrosis, and viral and bacterial infections [11,12,13]. Outstanding phagocytic capacity of these cells, usually considered as an unwanted particle clearance mechanism, can be exploited as a drug delivery strategy [14]. In the case of drug delivery to phagocytic cells, drug release should be possibly slowed down up to the moment of particle internalization. Application of slowly dissolving materials may be one of the strategies providing release delay. Another more robust way may be covalent bonding of drug molecule to the polymer matrix. Drug-tagged polymers preserve physical properties of pure polymers, and can be processed into various medical devices, i.a. particle-based drug delivery systems. Except for premature drug release prevention, such an approach ensures uniform drug distribution and drug loading depending less on particle preparation procedure. Amount of drug encapsulated in conjugate-based microspheres depends on molecular weight of drug-tagged polymer, whereas the amount of drug delivered to the particular phagocyte is determined by particle size and number of internalized particles. Therefore, in the described approach, particle size is a parameter responsible for internalization degree, particle distribution, and amount of delivered active pharmaceutical ingredient. In the context of macrophage-targeted drug delivery, particle diameter should be in the range of 1–3 μm preferred by phagocytes and determined i.a. by cell membrane curvature [15]. The targeting strategies vary according to aimed cell subsets and may require different diameters enabling blood-brain-barrier crossing, deep alveolar inhalation, and specific tissue accumulation. Therefore, along with a number of other features, particle cores should have desired size matched to therapeutic purpose.

### 1.3. Solvent Evaporation Technique

Solvent evaporation technique (SET) is one of the most frequently used techniques for polymeric particle production. This flexible technique enables incorporation of numerous active pharmaceutical ingredients in polymeric matrices and ensures desired release profiles [16]. Oil in water SET of particle preparation principle is based on evacuation of volatile organic phase from o/w emulsion, which results in solidification of water-insoluble particles. The final form of obtained drug carrier is affected primarily by emulsion properties. Possible variants and adjustable parameters of this well-established technique resulted in numerous investigations and reviews in past years [17,18]. SET modifications enabling more efficient drug loading were developed. Introduction of additional water phase in w/o/w SET enables incorporation of hydrophilic drugs [19]. Another approach of hydrophilic drug incorporation in form of suspension in organic phase was achieved in s/o/w SET [20].

Rotor-stator homogenizers are widely employed in laboratory-scale facilities. Homogenization parameters and quantitative and qualitative composition of homogenized two-phase systems influence the final shape and size of formed drug carriers. Choice of surfactant is highly important due to varying capability to prevent droplet coalescence and impact of stabilizing agent physiochemistry on surface charge of resulting particles [21]. Moreover, geometry of homogenizing tip, as well as volume and shape of homogenizer vessel influence emulsion droplet size. Shear stress and eddies were confirmed as important homogenization factors via in silico simulations [22]. Nevertheless, theoretical prediction of particle morphology is still surpassed by empirical evaluation of parameters and provides more reliable outcomes.

### 1.4. Evaluation of Lamivudine Conjugate-Based Particles

Lamivudine (LV), antiretroviral drug administered in human immunodeficiency virus type 1 infection was employed in the present study, as a model drug for conjugate synthesis. Recent findings underline the role of macrophages in human immunodeficiency virus (HIV) spreading and persistence mechanisms, especially in so-called “sanctuaries”, such as the central nervous system [23]. Thus, antiretroviral drugs could be delivered to infected macrophages as a part of complex therapy. Several attempts of LV conjugation were reported in recent years [24]. LV-dextran conjugate was synthesized via succinate linkers, obtained product exhibited altered biodistribution [25]. Modification of LV partition coefficient via ester linkage with stearic acid increased significantly drug loading in chitosan-based micelles [26]. Anti-HIV activity of phosphorylated LV derivatives conjugated with SiO_2_ particles was confirmed [27]. Poly (lactic-co-glycolic acid) (PLGA) was successfully conjugated with LV, and incorporated as nanoparticles in HIV-preventing thermosensitive vaginal gels [28]. LV loaded particles were obtained also via SET employing cellulose [29], Poly(3-hydroxybutirate-co-3-hydroxyvalerate) [30] or hydroxy-propyl cellulose [31]. Nevertheless, no attempts of conjugate-based particles obtained via SET were reported. In the present study, drug-initiated polymerization of cyclic monomer ε-caprolactone in ring opening polymerization (ROP) reaction followed by SET particle preparation for macrophage targeting applications was investigated. According to the suggested reaction mechanism of ROP, covalent bonding of hydroxyl bearing compound to polymeric chain occurs during initiation stage. Active pharmaceutical ingredient can be employed as initiator, resulting in drug-tagged polymer chains formation [32]. LV was chosen to confirm the possibility of hydrophilic drug incorporation in polymer matrix via SET without employing double emulsion approach, which frequently results in highly polydisperse particles [19]. Low molecular weight drug–polymer conjugate retains properties of pure polymer, thus may be processed in SET, and form spherical micro-matrices. The aim of the work was evaluation of the influence of o/w SET preparation parameters on the conjugate-based sphere diameter, including surfactant type, homogenization rate, homogenization time, phase ratio, surfactant concentration, polymer concentration in the dispersed phase.

## 2. Materials and Methods

### 2.1. Materials

Following materials were used in study: ε-caprolactone (purity 97%, Sigma Aldrich, Darmstadt, Germany) dried over calcium hydride caprolactone (purity 97%, Sigma Aldrich), distilled under reduced pressure prior use, stored under reduced pressure, lamivudine–secondary pharmaceutical standard (purity 100%, Sigma Aldrich), tin 2-ethylhexanoate (purity 92.5–100% Sigma Aldrich), Poly(vinyl alcohol) (31 kDA, degree of hydrolysis 86.7–88.7%, Roth, Zielona Góra, Poland), dichloromethane (purity 98.5%, Chempur, Piekary Śląskie, Poland), methanol (purity 99.5%, Chempur), CDCl_3_ (purity 100%, Sigma Aldrich), tetrahydrofuran (purity 99.8%, Chempur), polystyrene standards (Sigma Aldrich), acetonitrile (purity 99.9%, Sigma Aldrich), ammonium acetate (purity 97–100%, Chempur), glacial acetic acid (purity 99.5%, Chempur), sodium hydroxide (purity 98%, Chempur), hydrochloric acid (purity 99.9%, Chempur), polysorbate 80 (pharmaceutical grade, Biochemia Stosowana, Poznań, Poland), polyethylene glycol hexadecyl ether (PGHE) (pharmaceutical grade, Crode, Gouda, Netherlands), polystyrene standards (analytical standard grade, Sigma Aldrich).

### 2.2. Conjugate Synthesis

Bulk, drug-initiated ε-caprolactone (ε-CL) ring opening polymerization was performed as reported previously [33]. Briefly, mixture of LV, ε-CL and SO was stirred in 130 °C for 5 h in dry nitrogen atmosphere. Employed reactant ratio was as following: SO:LV:EC 0.1:2.8:87.7. Crude product was dissolved in dichloromethane and recrystallized from cold methanol, dried, characterized and stored in desiccator until further use.

### 2.3. Conjugate Degradation

In order to determine drug content in conjugate, material was exposed to elevated temperature and extreme pH to induce hydrolytic scission of polymer and drug-polymer bonds. Concentration of drug detached from conjugate was measured via HPLC. In order to determine most efficient conditions for accelerated degradation, 15 mg samples of conjugate were incubated in 10 mL of NaOH or HCL solutions of pH: 1.5, 2.5, 3.5, 8.7, 9.5, 11.8 for 21 days in 50 °C under mild shaking. Solution with pH 11.8 inducing most efficient conjugate degradation was chosen for degradation experiment in 100 °C with vigorous stirring. Samples were collected ten times in a period of 21 days in order to determine time necessary for hydrolytic scission of all drug-polymer bonds.

### 2.4. Particle Preparation

Various microsphere batches were obtained via o/w emulsion SET. Emulsion was obtained by homogenization of dichloromethane conjugate solution with distilled water surfactant solution with use of laboratory rotor-stator homogenizer X120 (Ingenieurbüro CAT, Ballrechten-Dottingen, Germany). Each phase was filtered prior use through 0.45 µm syringe filters, polytetrafluoroethylene filters (VWR, Radonor, PA, USA) were employed for dichloromethane phase filtration, and polyethersulfone filters (Milipore, Burlington, MA, USA) for aqueous phase filtration. Total emulsified volume was constant and equal 30 mL. Due to influence of vessel and rotor-stator geometry, centric position of homogenizer was maintained. Different homogenization parameters, oil/water phase ratios, surfactant and conjugate concentrations were employed in order to obtain various size of particles. After emulsification, samples were left under magnetic stirring to 350 rpm for 2 h in room temperature in order to evaporate dichloromethane. Parameters of each preparation are summarized in Table 1.

### 2.5. Power Consumption Measurement

The power consumption in homogenization vessel was determined by calorimetric measurements. Temperature increment in homogenized mixture kept in insulated vessel was recorded for different homogenization rates with use of thermocouple connected to digital multimeter (Tonghui, Changzhou, China). Thermocouple sensitivity was established in calibration procedure in range thermostated samples. The power was calculated according to equation determined by Oosterhuis and Kossen [34]. Mixture density was measured with pycnometric method, employed value of specific heat was 4.198 J × g^−1^ × K^−1^, measurements were performed for 30 mL of liquid.

### 2.6. Spectroscopic Analysis

The formation of the drug-polymer conjugates in ring opening polymerization was confirmed with electrospray mass spectrometry in acetonitrile on a micrOTOF-Q mass spectrometer (Bruker Daltonics, Bremen, Germany). Isotopic distribution of the peaks found by the experiment was compared to corresponding distributions of drug-tagged polymeric chains simulated by Mmass software. Proton nuclear magnetic resonance analysis (^1^H NMR) in CDCl_3_ was performed on Bruker 300 ultrashield NMR system (Bruker, Billerica, MA, USA).

### 2.7. Gel permation Chromatography

Number average molar mass (M_n_) of sample was determined with gel permeation chromatography (GPC). Chromatograms were obtained with use of Thermo Scientific high performance liquid chromatography (HPLC) set, Dionex Ultimate 3000 (Thermo Scientific, Waltham, MA, USA) equipped with Phenogel 10^3^A° column (Phenomenex, Torrance, CA, USA) in tetrahydrofuran, in room temperature. Obtained M_n_ values were based on polystyrene standard calibration, and adjusted with correcting coefficient equal to 0.56 [35].

### 2.8. High Performance Liquid Chromatography

LV concentrations in degradation study was measured with pharmacopoeial HPLC method [36]. Obtained solutions were filtered through 0.22 µm membranes and analyzed on Hitachi Primaide HPLC set (Hitachi HTA, Schaumburg, IL, USA), equipped with Purospher^®^STAR RP−18 endcapped (5 μm) 250 × 4.6 mm column (Merck Millipore, Burlington, MA, USA). Analysis was performed in 35 °C with 1 mL/min flow. 0.025 M Ammonium acetate solution, with pH adjusted to 3.8 ± 0.2 with acetic acid mixed with methanol in 95:5 ratio was employed as mobile phase. A Primaide 1410 UV detector (Hitachi HTA) was employed to detect analytes at 277 nm wavelength. Each sample was evaluated two times, reported concentrations are expressed as mean of duplicates.

### 2.9. Particle Size and Zeta Potential Evaluation

The size and morphology of two selected particle batches was investigated with scanning electron microscopy. Samples were coated with a fine gold layer and analyzed on a HELIOS NANOLAB 450HP microscope, 15 kV, (FEI, Hillsboro, OR, USA). Particle hydrodynamic diameter, polydispersity index (PDI) and zeta potential were evaluated via dynamic light scattering (DLS) measurements on Zetasizer Nano apparatus (Malvern, Worcestshire, UK), each sample was measured three times, hydrodynamic diameters are expressed as mean of triplicates.

## 3. Results

### 3.1. Conjugate Synthesis

**Figure 1:**
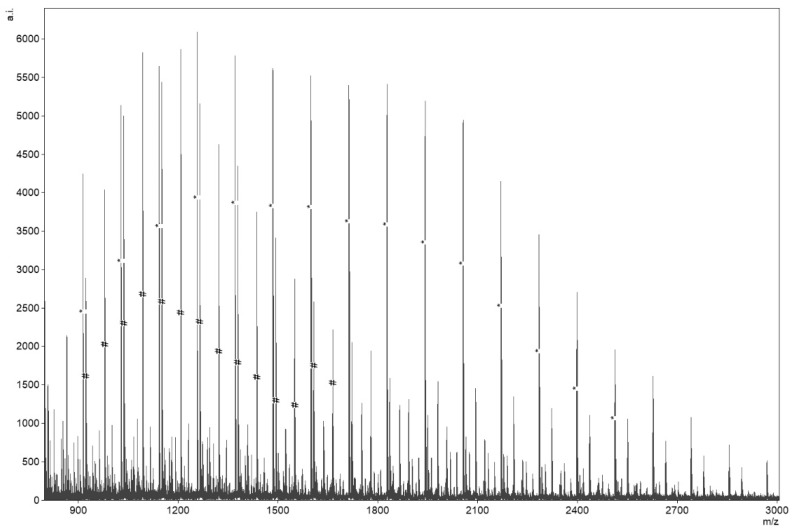
Mass spectra of synthesized lamivudine- poly-ε-caprolactone (LV–PCL) with doubly charged ions coupled with ammonium adducts (#) and singly charged ions coupled with protons (*).

Product of lamivudine-poly-ε-caprolactone conjugate (LV-PCL) synthesis was investigated with spectroscopic methods and GPC. Mass spectra of obtained product show characteristic for polymeric molecules distributions of peaks with peak sets separated from each other by 114 Da which is equal to molecular mass of poly-ε-caprolactone (PCL) monomer unit (Figure 1, *) or 57 Da in case of doubly charged ions (Figure 1, #). Isotopic distribution of peaks in two sets with highest intensities (Figure 1, *; #) matched isotopic distributions for LV-PCL simulated by software. Isotopic distribution simulations corresponded to respective experimental spectra for positively charged formula of LV-PCL conjugate with single positively charged hydrogen adduct and two positively charged ammonium adducts.

**Figure 2:**
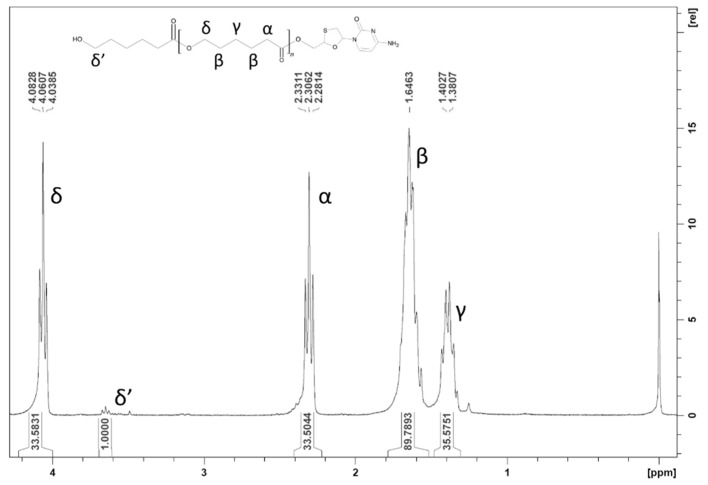
HNMR spectra of lamivudine-polycaprolactone conjugate.

Peaks observed on the ^1^H NMR spectra of the synthesized conjugate are as following: ^1^H NMR (300 MHz, CDCl_3_) δ = 4.06 (t, 2H, CH_2,_ δ), 3.64 (t, 2H, CH_2_, δ’), 2.30 (t, 2H, CH_2_, α), 1.65 (m, 2H, CH_2_, β), 1.38 (m, 2H, CH_2_, γ). Signals were assigned to individual protons (Figure 2). M_n_ derived from ratio between peak integrations of end group protons δ’ and repeating polymer group protons δ was equal 4.05 kDa.

Molecular weight of synthesized polymer molecules was evaluated with use of gel permeation chromatography. Obtained M_n_ relative to polystyrene standard was 7.14 kDa, with PDI 1.65. M_n_ value after adjustment according to correction coefficient was equal to 3.99 kDa, a value close to the one found by ^1^H NMR spectra.

### 3.2. Drug Content

**Figure 3:**
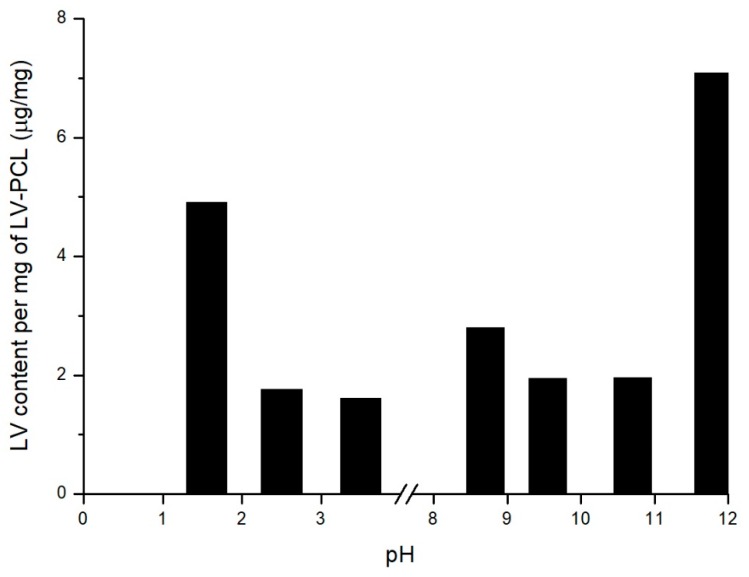
LV content in medium with varying pH after 21 days incubation in 50 °C.

**Figure 4:**
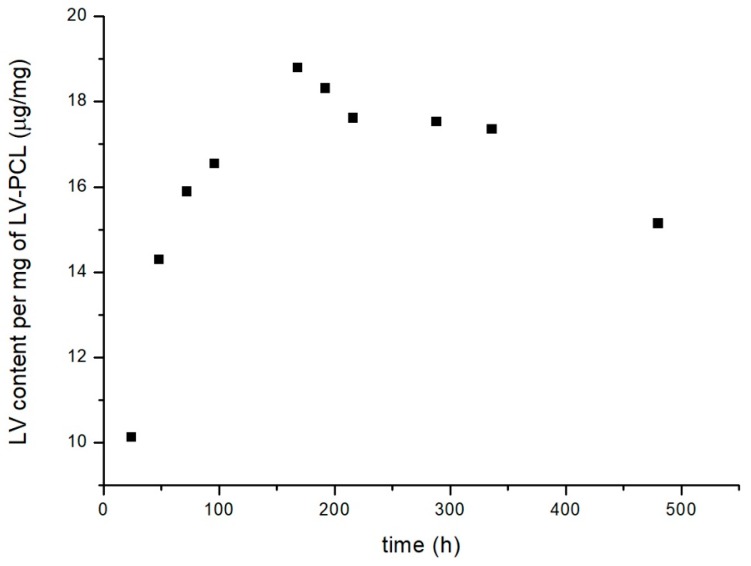
Amount of hydrolytically detached lamivudine per mg of conjugate at 100 °C, pH 11.8, within period of 21 days.

In order to estimate amount of drug fixed to polymer carrier, accelerated conjugate decomposition was induced by elevated temperature and pH modification. Most pronounced conjugate degradation followed by hydrolytic detachment of 7 μg of LV per 1 mg of LV-PCL was observed in pH of 11.8 (Figure 3). Efficient conjugate hydrolysis resulting in 5 μg of LV per 1 mg of LV-PCL was observed also at pH of 1.5. Further investigation in the most efficient accelerated degradation conditions (Figure 4) showed, that the highest drug concentration was observed in 7th day of experiment; the highest determined amount of LV was approximately 19 μg per 1 mg of conjugate. Further exposition of conjugate to pH of 11.8 in 100 °C resulted in decrease of LV concentration in the medium.

### 3.3. Particle Preparation

Particle suspensions obtained via SET were analyzed in light scattering experiments, measured hydrodynamic diameters were in range from 0.2 µm to 2.3 µm with polydispersity indexes in range from 0.19 up to 0.84. Homogenization rate, time and surfactant concentration shown correlation with particle hydrodynamic diameters. Zeta potentials of particles prepared with different surfactants varied. Particles obtained in preparations employing range of PVA concentration exhibited increase of zeta potential correlated with increasing surfactant content. Estimated power consumption during mixing at various homogenization rates was in the range of 6 W to 136 W. Measurement outcomes were summarized in Table 2.

**Figure 5:**
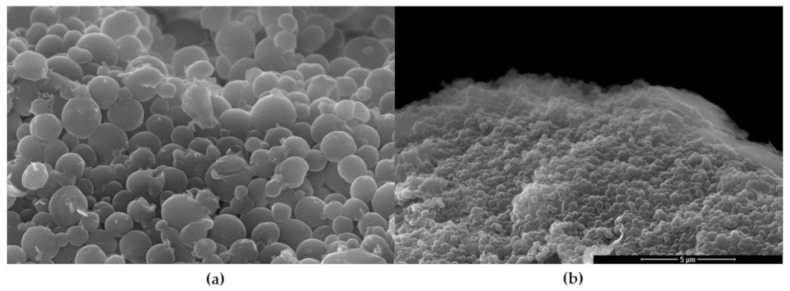
SEM micrographs of HS2 (**a**) and HS5 (**b**) preparations.

Scanning electron micrographs of HS2 and HS5 batches shows spherical structures with smooth surface. Particles observed in preparation HS2 (Figure 5a) have diameters ranging from 1.0 µm to 2.0 µm, and 0.2 µm–0.5 µm in preparation HS5 (Figure 5b).

## 4. Discussion

### 4.1. Conjugate Synthesis

Obtained mass spectra contain high average intensity isotopic peak distributions corresponding to simulations of LV-PCL ion isotopic distributions, which confirms coupling of LV with PCL chain. M_n_ of 4.17 kDa calculated from ^1^H NMR spectra, is nearly equal to corrected M_n_ of 3.99 kDa obtained from GPC analysis. M_n_ values obtained with the above methods are consistent with M_n_ of 3.77 kDa predicted from reactants ratio. This convergence confirms occurrence of ROP of monomer initiated by drug molecule and acceptable reaction control.

### 4.2. Drug Content

One of the key properties extensively evaluated in research focused on nano and micro particle drug delivery field is drug encapsulation efficiency [37]. In the present study, amount of medicinal substance in prepared particles, due to the covalent bonding between polymer and drug, depends on molecular weight of conjugate and size of particle. In order to accelerate conjugate degradation, elevated temperature and range of pH were applied. Extremely basic pH was found to be efficient degradation promoter. The drug incorporated into the carrier was hydrolytically detached from LV-PCL after 7 days. Final drug loading in material was estimated as ca. 19 μg per 1 mg of conjugate. Further exposition to high temperature and high pH resulted in decrease of LV concentration in medium, probably due to the degradation of drug in applied conditions [38]. The presented graph (Figure 4) reflects two concurrent processes: hydrolytic drug detachment from the conjugate, and drug degradation under high pH, thus the LV concentrations achievable in physiological conditions are expected to be higher and will be further studied.

### 4.3. Particle Formation and Morphology

**Figure 6:**
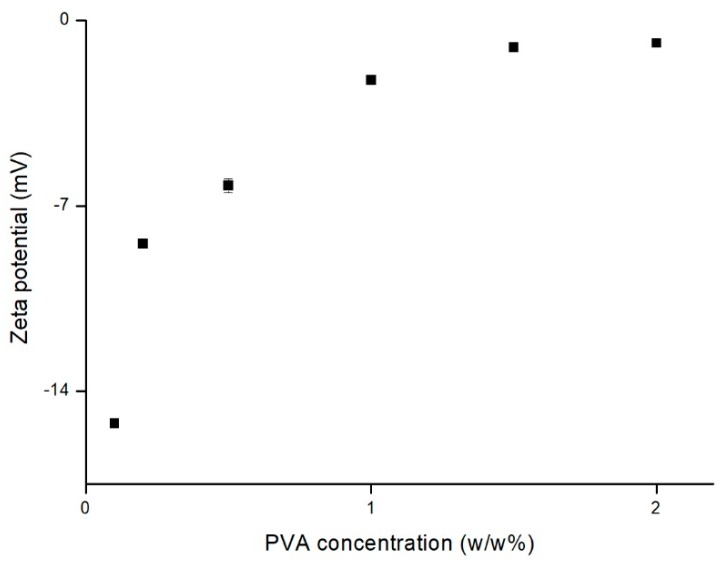
Influence of PVA concentration on the variability of particle zeta potential in preparations SC1-SC6, the bars represent SD.

Droplet breakup in rotor-stator homogenizers has been investigated both experimentally and theoretically. The emulsion parameters are crucial for the size of resulting particles produced via SET. The emulsion system is influenced by complex forces occurring in turbulent flow produced by high rate rotor-stator homogenizers. Numerous papers investigating influence of different parameters on size, PDI, shape, drug entrapment, and other micro and nano sized particles properties were published in recent decades [39]. This is due to specific effects of mentioned parameters on qualitative properties of various emerging formulations. The parameters must be suited for almost every new application in the field of drug delivery of medicinal substance. We consider it reasonable to compare outcomes within the applied method, as influence of preparation parameters is based on similar phenomena. The liquid dynamics responsible for dispersed droplet formation are described mainly within turbulent flow theory [40]. Eddies created during high shear mixing provide elongating forces, leading to droplet breakup. Complexity of forces occurring during homogenization hinders theoretical prediction of product properties, therefore particular systems are usually investigated experimentally. In this study, respective parameters were separately investigated, with other potentially influencing parameters maintained constant. The visualized HS2 and HS5 preparations, presented on SEM micrographs, consist of spherical particles with smooth surface (Figure 5). Diameter of the particles are in line with hydrodynamic diameter values obtained from DLS measurements. Zeta potential of particles obtained with polysorbate 80 was twofold higher in comparison to particles obtained with PVA and PGHE. High surface charge may be the result of relatively low polysorbate molecular weight and presence of hydrophobic chains, which allow adsorption of multiple molecules on lipophilic surface of particles. Number of polar hydroxyl and ester groups present in polysorbate 80 molecule contribute to the resulting particle zeta potential. Zeta potential of particles obtained in range of PVA concentrations (Figure 6) increased with increasing surfactant concentration. Similar observations were made during preparation of PLA and lipid particles with SET employing PVA [41,42]. In theory, more pronounced negative or positive zeta potential is beneficial in terms of colloid stability due to electrostatic repulsion between particles [43]. Therefore, lower PVA concentrations would be preferred in order to obtain stable particle suspensions.

**Figure 7:**
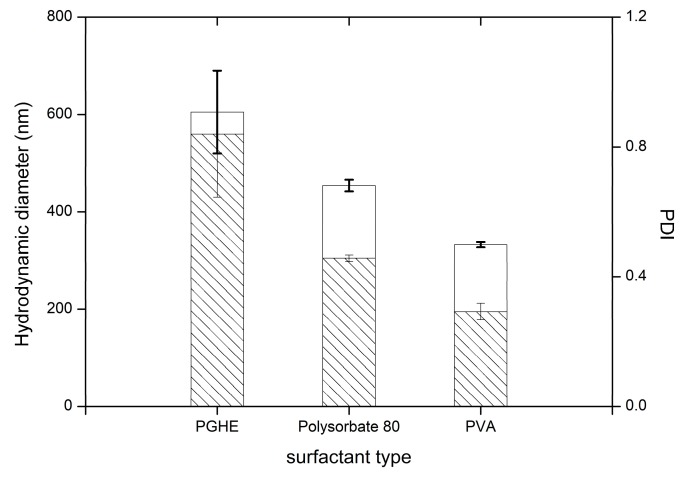
Influence of different surfactants on the variability of particle hydrodynamic diameter (□) and PDI (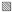) in preparations ST1-ST3, the bars represent SD.

Droplets formed during homogenization process, due to high surface area, tend to merge into larger structures to minimize the free energy. Addition of various stabilizers enables avoidance of this phenomena. In the presented work, the emulsions were stabilized using following non-ionic surfactants: Polysorbate 80, PGHE and PVA. Due to their low toxicity, stability and compatibility with pharmaceutical excipients, this group of surfactants is widely used in industrial applications [44]. Sorbitan derivative, Polysorbate 80 with 15 HLB number is a good candidate for stabilization of o/w formulations, including SET procedures, whereas the PVA is most frequently employed colloid stabilizer in particle preparation techniques. The PGHE is less frequently evaluated non-ionic surfactant, capable of o/w cream formulation stabilization [45,46]. Particles obtained in formulation stabilized by PVA exhibited significantly lower diameters, and narrow polydispersity (Figure 7). Due to low molecular weight, its solutions exhibit mild viscosity, which is beneficial in terms of chain mobility during interfacial orientation. Additionally, slight degree of acetylation hinders polymer chain-chain interaction but still enables interfacial adsorption. Slightly larger diameters and PDI were obtained with use of the Polysorbate 80 solutions, possibly due to their lower viscosity, comparing to PVA solutions. The molecular weight of employed Polysorbate 80 was approximately 30× lower comparing to PVA. The higher PVA solution viscosity could prevent coalescence of the emulsified droplets during the mixing [47]. Despite capability of PGHE to stabilize o/w viscous systems [48], particles obtained with use of it via SET were relatively large and highly polydisperse. Nevertheless, presented results confirm the possibility of obtaining sub-micron particles with the use of this emulsifier.

**Figure 8:**
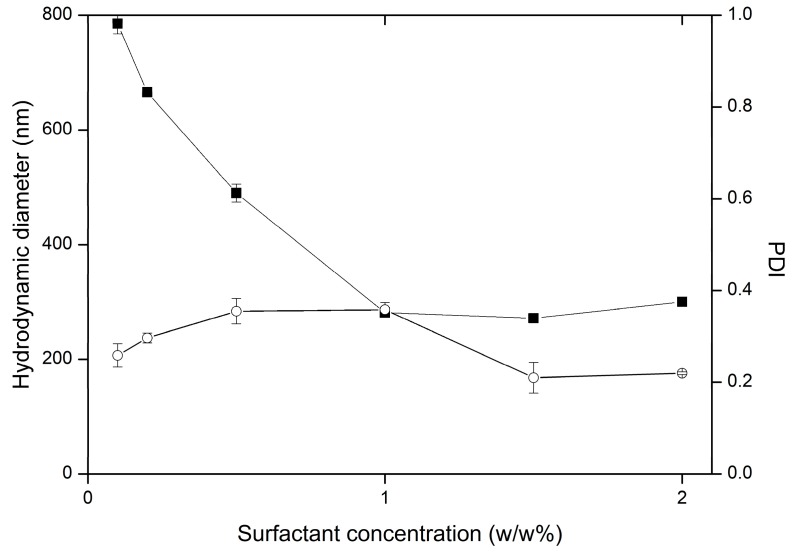
Influence of PVA concentration on the variability of particle hydrodynamic diameter (■) and PDI (○) in preparations SC1-SC6, the bars represent SD.

PVA was identified as the most efficient surfactant; impact of its concentration on particle hydrodynamic diameter was further evaluated. In line with expectations, stabilizer in higher concentrations was able to orient on larger interphase surface, and hence form stable structures with low diameters (Figure 8). Almost linear decrease of hydrodynamic diameter was observed when concentration increased from 0.02 *w*/*w*% to 1 *w*/*w*%. Further concentration increase did not influence particle size. Analogous effect was reported during albumin loaded poly(lactide-co-glycolide) submicron particle preparation. Diameter reduction from 63.1 μm to 0.14 μm was achieved with PVA increase from 0 to 0.9%, but further concentration increase had negligible impact on particle size [49]. Similarly, size of polyhydroxybutyrate-co-valerate microparticles could be reduced from 389 μm and 49 μm with PVA concentration increase from 0.5% to 1%, with no significant effect in higher concentrations [50]. Range of obtained diameters is in line with approximate emulsion drop size values of 0.2 μm–1.0 μm declared by surfactant manufacturer. Presumably, concentration about 1% percent results in most favorable viscosity in terms of colloid stabilization, and sufficient number of polymer molecules capable to adsorb interfacial on large surface.

**Figure 9:**
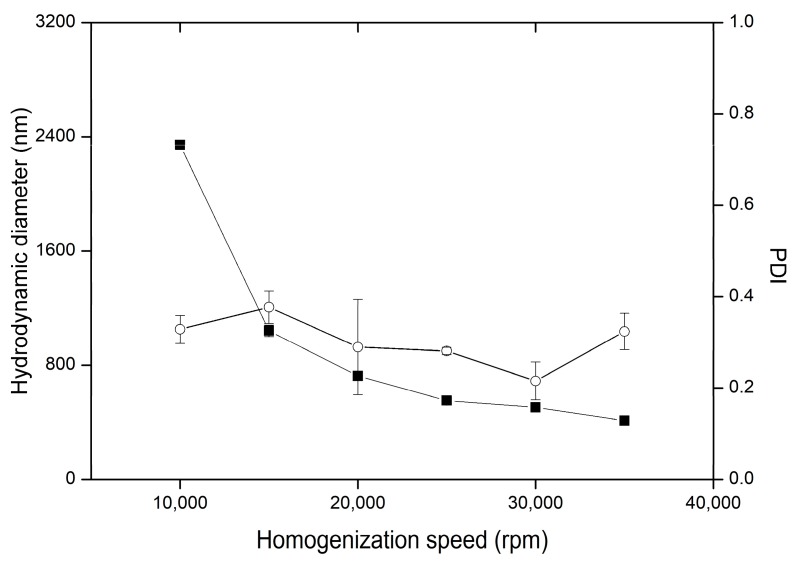
Influence of homogenization rate on the variability of hydrodynamic diameter (■) and PDI (○) in preparations HS1-HS6, the bars represent SD.

Increase of the homogenization rate, as in most of the reported works, effected in reduction of the diameter [51,52,53]. Such effect is linked to increased shear stress at faster rotation rates, which leads to larger tangential stress, and finally small emulsion droplets. In silico simulations of energy dissipation during rotor-stator homogenization suggest that two-folded increase of homogenization rate results in ca. eight-fold increase of energy dissipation [54]. In the present study, we observed particle diameter decrease exponentially with increase of homogenization rate (Figure 9). Decrease from micrometric to nanometric size was observed when homogenization rate exceeded 15,000 rpm. Further homogenization rate increases up to 35,000 rpm resulted in hydrodynamic diameter reduction below half of micron. Particle size dispersity was mildly fluctuating and was maintained in the range of 0.3 +/− 0.05.

**Figure 10:**
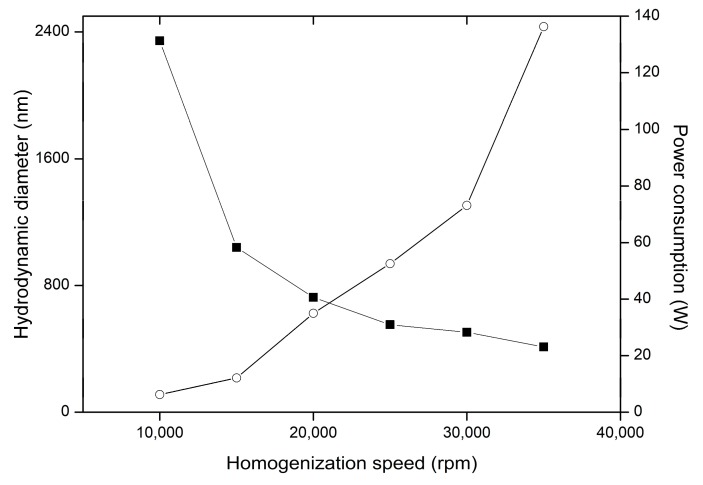
Influence of homogenization rate on the variability of particle hydrodynamic diameter (■) and power consumption (○) in preparations HS1-HS6.

Decrease of the particle diameter is correlated both with homogenization rate, as well as with power consumption increase (Figure 10). This result suggests vital contribution of overall energy introduced into system to the particle size. Power consumption is a parameter which may be measured in setups employing homogenizers with various rotor-stator geometries and different mixing vessels. The power consumption may reflect the size changes in homogenization processes, and thus it might be helpful in upscaling of the procedure.

**Figure 11:**
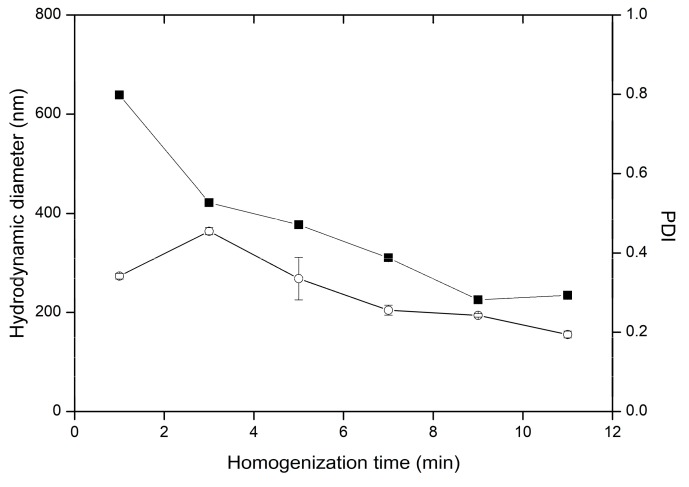
Influence of homogenization time on the variability of particle hydrodynamic diameter (■) and PDI (○) in preparations HT1-HT6, the bars represent SD.

The homogenizer worktime influenced particle hydrodynamic diameter, as well as particle polydispersity (Figure 11). The droplet breakup mechanism involves exposition of the droplets to the tangential stresses. It depends on employed homogenizer type and homogenizing vessel geometry and the value of rpm. The sufficient number of dispersed phase flow cycles through region between rotor and stator is required to achieve expected droplets size. The minimal particles diameter depends, however, on other parameters of the procedure and the evaluated composition. The most pronounced decrease in hydrodynamic diameter and PDI was observed during first nine minutes of homogenization. The time of mixing procedure favorable for formation of monodispersed particles is dictated by ratio between homogenized mixture volume and rotor-stator gap volume. Therefore, optimal mixing time should be determined for specific compositions, scales, and homogenizers.

**Figure 12:**
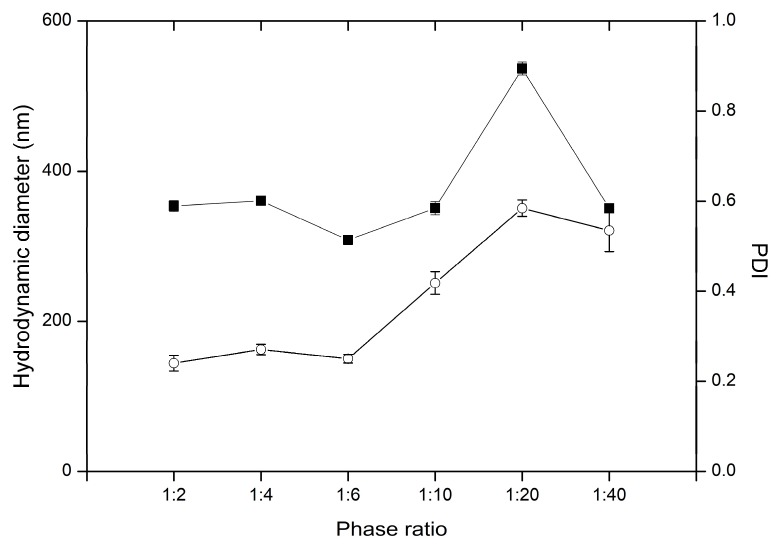
Influence of oil/water phase ratio on the variability of particle hydrodynamic diameter (■) and PDI (○) in preparations PR1-PR6, the bars represent SD.

The employed range of oil/water (o/w) phase ratios, did not impact the hydrodynamic diameter (Figure 12). According to available bibliography, the high volume of continuous phase may decrease the collision rate of particles, and subsequent coalescence [55]. Nonetheless, the results did not confirm any aggregation. The applied oil/water phase ratios provided stable two-hour phase of particle solidification, which resulted in monodisperse particle suspensions. On the contrary, particles were monodispersed when o/w ratio was low. In the preparations employing lower o/w ratios, with increased water content the overall viscosity of emulsion was higher: high viscous resisting forces may impair homogenization efficiency resulting in polydisperse particle suspensions [51]. The significant effect of oil volume fraction change on emulsion droplet diameter was reported both for heavy and light mineral oils, however the variations in low viscosity oil phase content did not influence droplet diameter [40]. Volatile organic phases employed in o/w SET are usually of low viscosity, therefore this parameter is not expected to significantly affect the diameter of obtained particles. The peak observed in ratio 1:20 may be attributed to unexpected factors of the process.

**Figure 13:**
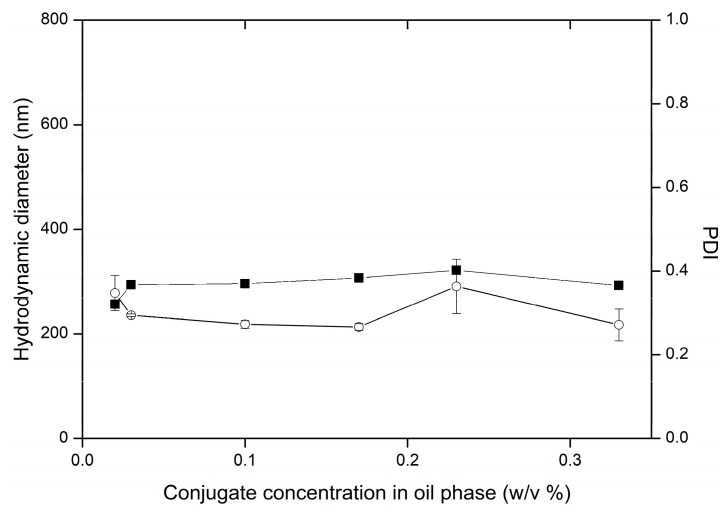
Influence of conjugate concentration on the variability of particle hydrodynamic diameter (■) and PDI (○) in preparations PM1-PM6, the bars represent SD.

Variability of the conjugate concentration dissolved in dispersed phase did not influence the hydrodynamic diameter, neither the PDI (Figure 13), although the high polymer concentration results in increased viscosity of dispersed phase [37]. Similarly, as above mentioned, the high resisting viscous forces during homogenization should lead to formation of high-sized oil droplets, and respective particles. Increase in diameter from 0.4 μm to 1.0 μm associated with respective increase of polymer concentration in dispersed phase from 10% to 20% was reported for PLGA microparticles [51]. Presumably, the applied polymer concentration range increased the viscosity inadequately, and did not influence sufficiently the droplet breakup.

## 5. Conclusions

Obtained lamivudine-tagged polycaprolactone can be used in potential targeted drug delivery systems. Micro- and nano- spheres obtained by solvent evaporation method had spherical shape, smooth surface and size of 0.2 μm to 2.4 μm. Covalent bonding between drug and polymer ensures an even distribution of drug and eliminates problems related to drug entrapment in polymer matrix. Among evaluated preparation parameters, homogenization rate, homogenization time, and surfactant concentration were identified as crucial parameters influencing particle hydrodynamic diameter. Obtained results may be helpful during design of size-dependent sub-micron drug delivery systems.

## Figures and Tables

**Table 1:** Particle preparation parameters employed in solvent evaporation method.

Batch	Homogenization Rate (rpm)	Homogenization Time (min)	Surfactant Concentration (*w*/*w*%)	o/w Phase Ratio	Surfactant Type	Polymer Concentration (*w*/*v*%)
ST1	25,000	5	0.5	1:10	PGHE	0.17
ST2	25,000	5	0.5	1:10	Polysorbate 80	0.17
ST3	25,000	5	0.5	1:10	PVA	0.17
SC1	25,000	5	0.1	1:10	PVA	0.17
SC2	25,000	5	0.2	1:10	PVA	0.17
SC3	25,000	5	0.5	1:10	PVA	0.17
SC4	25,000	5	1	1:10	PVA	0.17
SC5	25 000	5	1.5	1:10	PVA	0.17
SC6	25,000	5	2	1:10	PVA	0.17
HS1	10,000	5	1	1:10	PVA	0.17
HS2	15,000	5	1	1:10	PVA	0.17
HS3	20,000	5	1	1:10	PVA	0.17
HS4	25,000	5	1	1:10	PVA	0.17
HS5	30,000	5	1	1:10	PVA	0.17
HS6	35,000	5	1	1:10	PVA	0.17
HT1	25,000	1	0.5	1:10	PVA	0.17
HT2	25,000	3	0.5	1:10	PVA	0.17
HT3	25,000	5	0.5	1:10	PVA	0.17
HT4	25,000	7	0.5	1:10	PVA	0.17
HT5	25,000	9	0.5	1:10	PVA	0.17
HT6	25,000	11	0.5	1:10	PVA	0.17
PR1	25,000	5	0.5	1:2	PVA	0.17
PR2	25,000	5	0.5	1:4	PVA	0.17
PR3	25,000	5	0.5	1:6	PVA	0.17
PR4	25,000	5	0.5	1:10	PVA	0.17
PR5	25,000	5	0.5	1:20	PVA	0.17
PR6	25,000	5	0.5	1:40	PVA	0.17
CM1	25,000	5	0.5	1:10	PVA	0.02
CM2	25,000	5	0.5	1:10	PVA	0.03
CM3	25,000	5	0.5	1:10	PVA	0.10
CM4	25,000	5	0.5	1:10	PVA	0.17
CM5	25,000	5	0.5	1:10	PVA	0.23
CM6	25,000	5	0.5	1:10	PVA	0.33

**Table 2:** Summary of particle hydrodynamic diameter and polydispersity index (PDI) obtained in all preparations.

Batch	Hydrodynamic Diameter ± RSD (nm)	PDI ± RSD	Zeta Potential ± RSD (mV)
ST1	605 ± 14.05%	0.84 ± 23.03%	−6.89 ± 2.51%
ST2	454 ± 2.65%	0.46 ± 2.22%	−17.77 ± 15.52%
ST3	332 ± 1.63%	0.29 ± 8.74%	−8.57 ± 4.00%
SC1	785 ± 2.22%	0.26 ± 9.84%	−15.2 ± 1.14%
SC2	666 ± 1.11%	0.3 ± 3.61%	−8.43 ± 1.96%
SC3	490 ± 3.17%	0.35 ± 7.83%	−6.23 ± 4.36%
SC4	282 ± 1.04%	0.36 ± 4.33%	−2.26 ± 7.12%
SC5	272 ± 1.59%	0.21 ± 16.04%	−1.02 ± 10.01%
SC6	300 ± 1.33%	0.22 ± 1.39%	−0.85 ± 9.88%
HS1	2344 ± 1.41%	0.33 ± 9.24%	-
HS2	1041 ± 3.92%	0.38 ± 9.49%	-
HS3	725 ± 4.31%	0.29 ± 35.96%	-
HS4	552 ± 2.18%	0.28 ± 2.78%	-
HS5	505 ± 1.94%	0.22 ± 19.21%	-
HS6	412 ± 4.04%	0.32 ± 12.31%	-
HT1	639 ± 0.54%	0.34 ± 2.08%	-
HT2	422 ± 1.43%	0.46 ± 1.95%	-
HT3	377 ± 2.22%	0.34 ± 16.01%	-
HT4	310 ± 2.16%	0.26 ± 4.89%	-
HT5	226 ± 1.58%	0.24 ± 1.86%	-
HT6	235 ± 1.86%	0.19 ± 4.38%	-
PR1	354 ± 2%	0.24 ± 7.27%	-
PR2	361 ± 1.54%	0.27 ± 4.26%	-
PR3	308 ± 0.74%	0.25 ± 3.8%	-
PR4	351 ± 2.49%	0.42 ± 6.02%	-
PR5	537 ± 1.58%	0.58 ± 3.1%	-
PR6	351 ± 0.36%	0.54 ± 8.66%	-
PM1	257 ± 1.89%	0.35 ± 12.08%	-
PM2	294 ± 0.86%	0.29 ± 1.09%	-
PM3	296 ± 0.26%	0.27 ± 3.49%	-
PM4	307 ± 0.61%	0.27 ± 3.01%	-
PM5	322 ± 2.35%	0.36 ± 18.08%	-
PM6	293 ± 1.36%	0.27 ± 13.94%	-
